# A Cardiopulmonary Exercise Testing-Based Assessment: Can Wearable-Assisted Cardiac Rehabilitation Improve Functional Capacity in Patients With Chronic Heart Failure?

**DOI:** 10.7759/cureus.114974

**Published:** 2026-08-22

**Authors:** Mohamed Ztati, Wissame Dahmane, El Karimi Saloua, Mustapha El Hattaoui

**Affiliations:** 1 Cardiology, Mohammed VI University Hospital Center, Faculty of Medicine and Pharmacy of Marrakech (FMPM), Cadi Ayyad University, Marrakech, MAR; 2 Interventional Cardiology, Mohammed VI University Hospital Center, Faculty of Medicine and Pharmacy of Marrakech (FMPM), Cadi Ayyad University, Marrakech, MAR

**Keywords:** cardiac rehabilitation, cardiopulmonary exercise testing, functional capacity, heart failure, telerehabilitation, vo₂peak, wearable devices

## Abstract

Background

Cardiac rehabilitation is a cornerstone of chronic heart failure management and has consistently been associated with enhanced exercise performance and better health-related quality of life. Despite these benefits, access to conventional center-based rehabilitation remains limited in many low-resource settings. Wearable technologies offer the possibility of extending rehabilitation beyond specialized facilities through continuous physiological monitoring and individualized exercise guidance.

Objective

This study aimed to compare the effects of wearable-assisted and conventional cardiac rehabilitation on functional capacity evaluated through cardiopulmonary exercise testing (CPET) among individuals with chronic heart failure.

Methods

This prospective randomized monocentric study included patients with chronic heart failure enrolled in a structured 12-week cardiac rehabilitation program between January 2025 and February 2026. A total of 143 patients were randomized to wearable-assisted rehabilitation (n = 66) or conventional rehabilitation (n = 77). Twenty-two patients did not complete the follow-up assessment, resulting in a final analysis population of 121 patients: 54 in the wearable-assisted group and 67 in the conventional rehabilitation group. Functional capacity was assessed before and after rehabilitation using CPET-derived parameters, including peak oxygen consumption (VO₂peak) and first ventilatory threshold (SV1).

Results

The study population exhibited marked baseline exercise limitation, with a mean VO₂peak of 11.75 ± 1.36 mL/kg/min and a mean SV1 of 9.92 ± 1.11 mL/kg/min. Following rehabilitation, substantial physiological improvements were observed across the entire cohort. The mean VO₂peak increased to 16.75 ± 1.01 mL/kg/min (p < 0.001), corresponding to an average gain of approximately 5 mL/kg/min. The mean SV1 increased to 11.92 ± 0.86 mL/kg/min (p < 0.001), reflecting improved aerobic efficiency and delayed onset of anaerobic metabolism.

When rehabilitation modalities were analyzed separately, functional gains were remarkably similar. Patients undergoing wearable-assisted rehabilitation demonstrated a mean VO₂peak increase of 5.01 mL/kg/min compared with 5.00 mL/kg/min in the conventional rehabilitation group (p = 0.902). Improvements in SV1 were likewise comparable between groups (+2.02 vs. +2.00 mL/kg/min; p = 0.902).

Conclusions

Cardiac rehabilitation resulted in meaningful gains in exercise capacity in individuals with chronic heart failure. Wearable-assisted rehabilitation achieved physiological benefits comparable to those observed with conventional rehabilitation, supporting its potential as a practical alternative in settings where access to specialized rehabilitation facilities is limited.

## Introduction

Heart failure remains a major public health challenge and is associated with substantial morbidity, recurrent hospitalizations, impaired quality of life, and increased mortality. Beyond its clinical consequences, heart failure is characterized by marked reductions in functional capacity that frequently limit patients' ability to perform daily activities and contribute significantly to disease burden [1,2]. Cardiopulmonary exercise testing (CPET) is widely regarded as the gold-standard technique for assessing exercise performance and functional limitation in patients with heart failure. Among CPET-derived variables, peak oxygen consumption (VO₂peak) is a robust marker of functional impairment and prognosis, while the first ventilatory threshold (SV1) provides complementary information regarding submaximal exercise performance and aerobic efficiency [3-6].

Cardiac rehabilitation is a fundamental component of contemporary heart failure management. Structured exercise training has consistently been shown to improve exercise tolerance, symptoms, and health-related quality of life and is therefore strongly recommended as part of comprehensive heart failure care [7-9]. However, participation in conventional center-based rehabilitation remains limited in many regions, particularly in low- and middle-income countries. Limited availability of specialized facilities, transportation difficulties, geographic distance, and financial constraints may substantially restrict access to rehabilitation. In addition, social and socioeconomic determinants, including income, educational level, employment conditions, housing and food security, and access to healthcare, are increasingly recognized as important determinants of cardiovascular health and may further contribute to disparities in access to preventive and rehabilitative care [10]. These factors are particularly relevant when considering rehabilitation strategies that require repeated attendance at specialized healthcare facilities.

Wearable technologies may provide an opportunity to address some of these barriers by extending cardiac rehabilitation beyond the hospital setting. By enabling continuous heart-rate monitoring and individualized regulation of exercise intensity, wearable devices may facilitate remote supervision while preserving the physiological principles of structured exercise training. Such approaches may be particularly relevant for patients facing geographic, transportation, financial, or social barriers to conventional rehabilitation. Nevertheless, evidence evaluating wearable-assisted cardiac rehabilitation using objective CPET-derived outcomes remains limited, particularly in North African populations.

The present study aimed to compare the effects of wearable-assisted and conventional cardiac rehabilitation on functional capacity, assessed using CPET, among individuals with chronic heart failure. We specifically evaluated changes in VO₂peak and SV1 following a structured 12-week rehabilitation program.

## Materials and methods

Study setting and participant selection

This prospective randomized monocentric study was conducted at the Cardiology Department of Mohammed VI University Hospital Center in Marrakech, Morocco, between January 2025 and February 2026. Consecutive patients with chronic heart failure referred for a structured cardiac rehabilitation program were assessed for study participation. Ethical approval was obtained from the Institutional Ethics Committee of Mohammed VI University Hospital (approval number: EC-M6UH-2025-001).

The study population consisted of ambulatory patients with chronic heart failure referred for phase II cardiac rehabilitation after clinical stabilization. Baseline demographic and clinical characteristics, including age, sex, body mass index (BMI), New York Heart Association (NYHA) functional class, left ventricular ejection fraction (LVEF), and CPET-derived parameters, were collected for all participants before rehabilitation.

Patients were eligible for inclusion if they were adults with clinically stable chronic heart failure with reduced or mildly reduced LVEF, according to the 2021 European Society of Cardiology (ESC) guidelines [5]. Heart failure with reduced ejection fraction (HFrEF) was defined as an LVEF ≤40%, whereas heart failure with mildly reduced ejection fraction (HFmrEF) was defined as an LVEF of 41-49% [5]. Patients were also required to remain symptomatic despite guideline-directed medical therapy, to be considered suitable for participation in a structured cardiac rehabilitation program after exclusion of contraindications to exercise training, and to be able to undergo CPET both at baseline and after the completion of the rehabilitation program.

Patients were excluded if they had contraindications to exercise-based cardiac rehabilitation or CPET, including acute decompensated heart failure, recent acute coronary syndrome, uncontrolled cardiac arrhythmias, severe symptomatic valvular heart disease, uncontrolled hypertension, or severe musculoskeletal or neurological disorders limiting safe exercise participation.

Eligible patients were randomized in a 1:1 ratio using a computer-generated randomization sequence prepared in Microsoft Excel (Microsoft Corp., Redmond, WA, USA). At enrollment, each participant was assigned a study number corresponding to the pre-generated randomization list, which determined allocation to either the wearable-assisted or conventional rehabilitation group. A total of 143 patients were randomized before the initiation of the rehabilitation program, with 66 assigned to the wearable-assisted group and 77 to the conventional rehabilitation group. Patients who subsequently died, were lost to follow-up, or were unable to complete the assigned rehabilitation strategy were not included in the final per-protocol analysis.

Cardiac rehabilitation program

All patients participated in a structured 12-week cardiac rehabilitation program, with exercise prescriptions individualized according to baseline clinical evaluation and CPET findings. Participants were randomly allocated to one of two rehabilitation strategies.

The wearable-assisted rehabilitation group performed three home-based aerobic training sessions per week, with each session lasting approximately 30-45 minutes. Activities were individualized according to functional capacity and included walking, active or brisk walking, cycling, and combined walking and cycling. Exercise intensity was prescribed using the Karvonen method, with a target heart rate corresponding to 50-70% of heart-rate reserve (HRR), calculated as follows: $\text{target HR}=\text{resting HR}+[0.50-0.70\times(\text{peak HR}-\text{resting HR})]$. Heart rate was monitored using an Apple Watch Series 10 (Apple Inc., Cupertino, CA, USA) during home-based sessions. The target heart-rate range was established at baseline and remained unchanged throughout the 12-week rehabilitation program; no repeat CPET was performed for the purpose of adjusting exercise intensity.

Patients in the conventional rehabilitation group underwent standard center-based rehabilitation sessions supervised by the rehabilitation team according to institutional practice. The exercise objective in both groups was to achieve sustained moderate-intensity aerobic exercise while ensuring safety and adherence. Patients in the wearable-assisted group underwent clinical follow-up approximately once monthly, during which symptoms, exercise tolerance, and clinical improvement were assessed. Adjustments to the rehabilitation program were made when clinically indicated, although the prescribed target heart-rate range was not routinely modified during the 12-week intervention.

CPET

Functional capacity was assessed using a graded cardiopulmonary exercise test conducted on a bicycle ergometer before rehabilitation initiation and repeated at the end of the 12-week program.

The principal CPET variables analyzed were VO₂peak and SV1. Additional parameters included ventilatory efficiency, oxygen pulse, respiratory exchange ratio, and peak heart rate. VO₂peak was considered the primary marker of global functional capacity, whereas SV1 was used to assess submaximal exercise performance and aerobic efficiency.

Study parameters

Baseline demographic, clinical, echocardiographic, and CPET variables were collected for all participants. Demographic variables included age, sex, and BMI. Clinical assessment included NYHA functional class, resting heart rate, and pulmonary artery systolic pressure (PASP). Echocardiographic evaluation included LVEF. CPET-derived parameters included VO₂peak, percentage of predicted VO₂peak, SV1, ventilatory efficiency (ventilatory equivalent for carbon dioxide (VE/VCO₂) slope), respiratory exchange ratio, oxygen pulse, and training heart rate. Information regarding exercise intensity, type of physical activity performed during the rehabilitation program, and Minnesota Living with Heart Failure Questionnaire scores before and after rehabilitation were also recorded. The primary outcome measure was the change in VO₂peak following the completion of the rehabilitation program, while secondary outcome measures included changes in SV1 and comparison of functional improvements between the wearable-assisted and conventional rehabilitation groups.

Study endpoints

The primary endpoint was the change in VO₂peak following the completion of the rehabilitation program. Secondary endpoints included changes in SV1 and comparative evaluation of functional improvements between wearable-assisted and conventional rehabilitation strategies.

Sample size

As this was a prospective single-center study, all consecutive patients with chronic heart failure who met the eligibility criteria and were referred for cardiac rehabilitation between January 2025 and February 2026 were included. Therefore, no a priori sample size calculation was performed, and the final sample size was determined by the number of eligible patients enrolled during the study period. A post hoc power analysis based on the primary endpoint (change in VO₂peak) demonstrated that the final sample of 121 patients provided a statistical power greater than 99% at a two-sided α level of 0.05, supporting the adequacy of the study sample to detect the observed treatment effect.

Statistical analysis

Quantitative data are presented as mean ± standard deviation, whereas qualitative variables are reported as counts and proportions. Continuous variables between the rehabilitation groups were compared using the independent Student's t-test, whereas categorical variables were compared using the chi-squared test. Pre- and post-rehabilitation CPET variables were compared using the paired Student's t-test to evaluate functional changes over time. Comparative analyses between wearable-assisted and conventional rehabilitation groups were performed to assess potential differences in rehabilitation-related gains.

Statistical significance was defined by a two-tailed p-value below 0.05. Data processing and statistical analyses were conducted using IBM SPSS Statistics for Windows, V. 26.0 (IBM Corp., Armonk, NY, USA).

## Results

Study population

**Figure 1:**
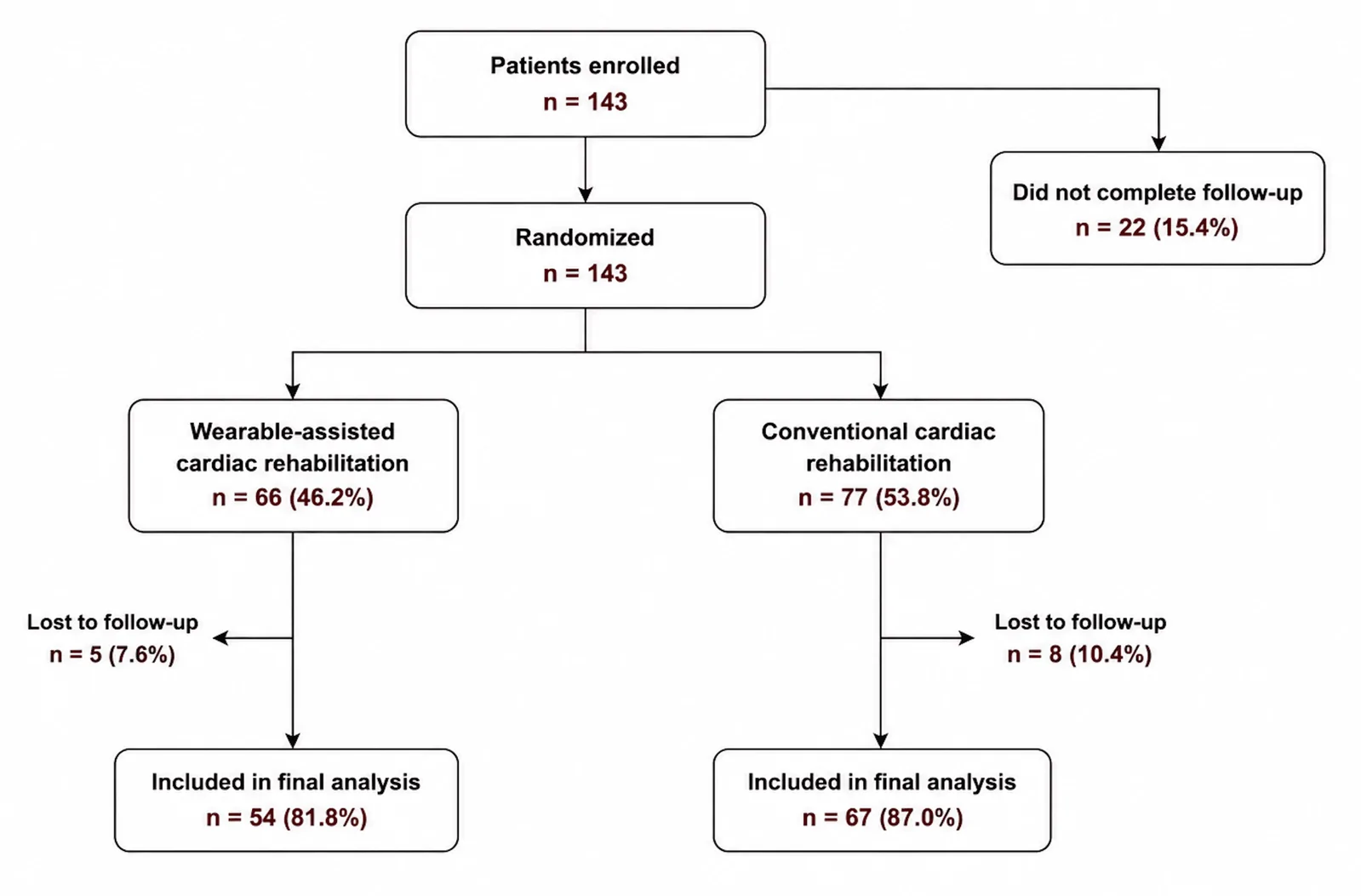
Study flow diagram showing randomization, follow-up, reasons for non-completion, and final analysis according to rehabilitation strategy

A total of 143 patients with chronic heart failure were randomized to either wearable-assisted rehabilitation (n = 66) or conventional rehabilitation (n = 77). During the follow-up period, 22 patients (15.4%) did not complete the predefined follow-up assessment and were therefore not included in the final analysis. Among these, 13 patients (59.1%) were lost to follow-up, including five (7.6%) from the wearable-assisted group and eight (10.4%) from the conventional rehabilitation group. Six patients (27.3% of those not completing follow-up), all assigned to the wearable-assisted group, were unable to obtain the wearable device because of financial constraints, representing 9.1% of the wearable-assisted group. Three patients (13.6%) died during follow-up, including one (1.5%) from the wearable-assisted group and two (2.6%) from the conventional rehabilitation group. The final analysis therefore included 121 patients who completed the rehabilitation program and had both baseline and follow-up CPET assessments: 54 in the wearable-assisted group and 67 in the conventional rehabilitation group. The study flowchart is presented in Figure 1.

Baseline characteristics

Table 1 presents the baseline demographic and functional profile of the study population. No meaningful differences were observed between the rehabilitation groups at enrollment. The mean age was 67.33 ± 4.18 years in the wearable-assisted rehabilitation group and 68.30 ± 3.98 years in the conventional rehabilitation group (p = 0.963).

**Table 1:** Baseline characteristics according to rehabilitation strategy Continuous variables were compared using Student's t-test and categorical variables using the chi-squared test. Statistical significance was defined as p < 0.05. BMI: body mass index; NYHA: New York Heart Association; VO₂peak: peak oxygen consumption; SV1: first ventilatory threshold; LVEF: left ventricular ejection fraction

Variable	Wearable	Conventional	P-value
Age	67.33 ± 4.18	68.30 ± 3.98	0.195
Sex (overall)			
Female sex	22 (40.7%)	29 (43.3%)	0.778
Male sex	32 (59.3%)	38 (56.7%)	
NYHA class (overall)			
NYHA II	8 (14.8%)	12 (17.9%)	0.892
NYHA III	32 (59.3%)	39 (58.2%)	
NYHA IV	14 (25.9%)	16 (23.9%)	
LVEF category (overall)			
LVEF 20-25%	5 (9.3%)	5 (7.5%)	0.987
LVEF 30-35%	27 (50.0%)	34 (50.7%)	
LVEF 35-40%	14 (25.9%)	16 (23.9%)	
LVEF 41-45%	4 (7.4%)	6 (9.0%)	
LVEF 45-49%	4 (7.4%)	6 (9.0%)	
BMI category (overall)			
BMI <18.5	11 (20.4%)	14 (20.9%)	0.608
BMI 18.5-25	1 (1.9%)	0 (0.0%)	
BMI 25-30	15 (27.8%)	25 (37.3%)	
BMI 30-40	10 (18.5%)	12 (17.9%)	
BMI >40	17 (31.5%)	16 (23.9%)	
VO₂peak baseline	11.69 ± 1.40	11.79 ± 1.34	0.690
SV1 baseline	9.85 ± 1.12	9.96 ± 1.12	0.592

Functional impairment was already evident at baseline. The mean VO₂peak was 11.69 mL/kg/min in the wearable-assisted rehabilitation group and 11.79 mL/kg/min in the conventional rehabilitation group (p = 0.674). Similarly, baseline SV1 values were comparable between groups (9.85 vs. 9.96 mL/kg/min; p = 0.615). Baseline heart failure medical therapy was also comparable between the two groups, with no significant differences in the use of guideline-directed medical therapies. No significant differences were observed between rehabilitation strategies before intervention.

Effect of cardiac rehabilitation on functional capacity

Completion of the rehabilitation program was associated with marked improvements in exercise performance across the entire cohort.

**Figure 2:**
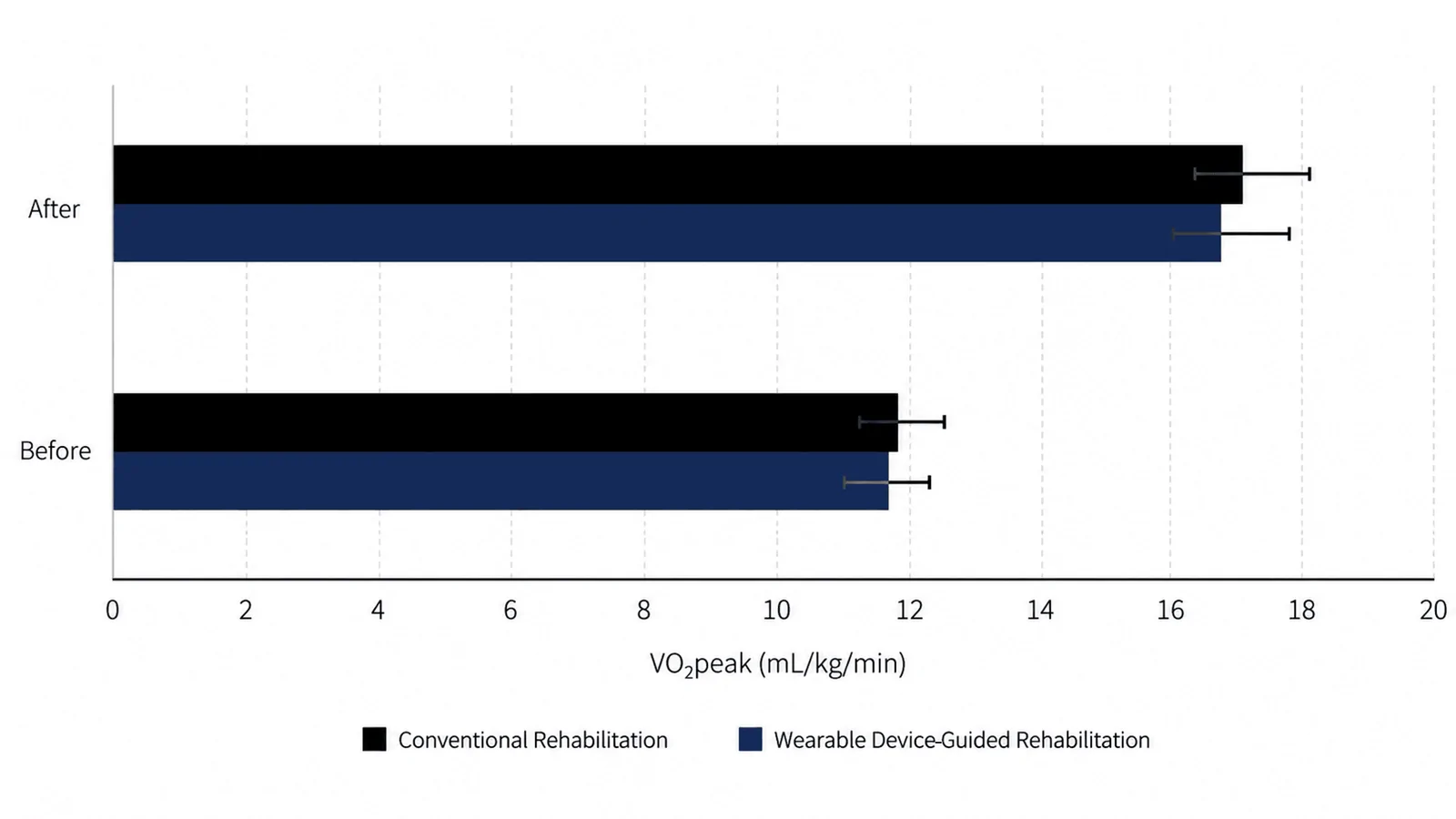
VO₂peak before and after rehabilitation in the wearable-assisted and conventional groups VO₂peak: peak oxygen consumption

The mean VO₂peak increased from 11.75 ± 1.36 to 16.75 ± 1.01 mL/kg/min (p < 0.001), corresponding to an average gain of approximately 5 mL/kg/min. This improvement reflects a substantial increase in aerobic exercise capacity in a population characterized by severe baseline functional limitation. The evolution of VO₂peak according to rehabilitation strategy is illustrated in Figure 2.

A parallel improvement was observed for SV1. The mean SV1 increased from 9.92 ± 1.11 to 11.92 ± 0.86 mL/kg/min (p < 0.001), indicating enhanced aerobic efficiency and a delayed transition toward anaerobic metabolism during exercise. Changes in CPET parameters before and after rehabilitation are summarized in Table 2.

**Table 2:** Changes in cardiopulmonary exercise testing parameters in the whole cohort Pre- and post-rehabilitation values were compared using the paired Student's t-test. Statistical significance was defined as p < 0.05. VO₂peak: peak oxygen consumption; SV1: first ventilatory threshold

Variable	Baseline	Post-rehabilitation	P-value
VO₂peak (mL/kg/min), mean ± SD	11.75 ± 1.36	16.75 ± 1.01	<0.001
SV1 (mL/kg/min), mean ± SD	9.92 ± 1.11	11.92 ± 0.86	<0.001

Wearable-assisted versus conventional rehabilitation

**Figure 3:**
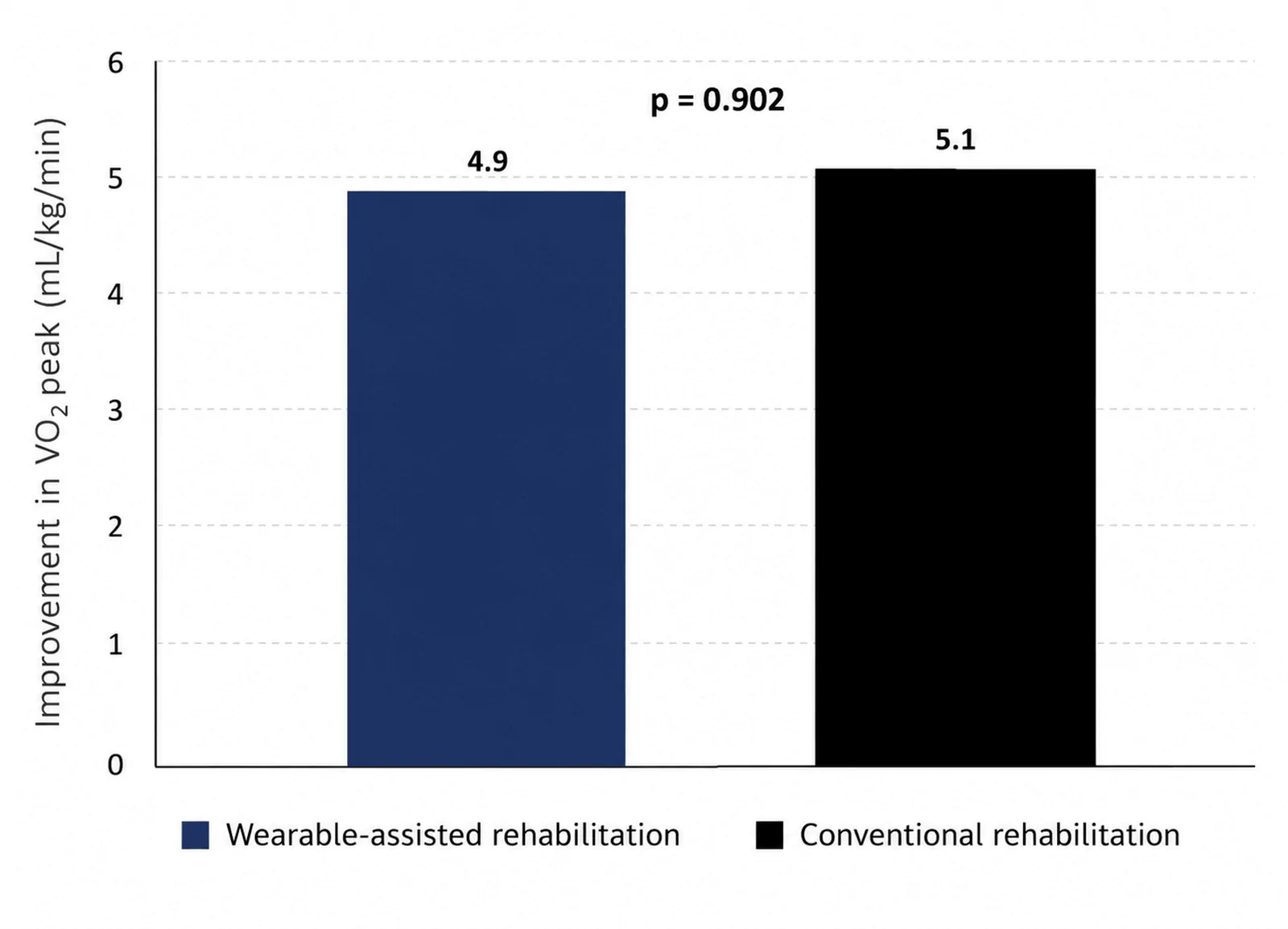
Mean improvement in peak oxygen consumption (ΔVO₂peak) according to rehabilitation strategy. No significant difference was observed between wearable-assisted and conventional rehabilitation (p = 0.902)

Analysis according to rehabilitation modality demonstrated remarkably similar physiological adaptations. Patients undergoing wearable-assisted rehabilitation exhibited an increase in VO₂peak from 11.69 to 16.70 mL/kg/min, corresponding to a mean gain of 5.01 mL/kg/min. Patients participating in conventional rehabilitation demonstrated an increase from 11.79 to 16.79 mL/kg/min, corresponding to a mean gain of 5.00 mL/kg/min. No significant difference was observed between groups regarding VO₂peak improvement (p = 0.902). Comparative improvements in VO₂peak are illustrated in Figure 3.

Likewise, SV1 increased by 2.02 mL/kg/min in the wearable-assisted rehabilitation group and by 2.00 mL/kg/min in the conventional rehabilitation group. The magnitude of improvement did not differ significantly between rehabilitation strategies (p = 0.902). Comparative functional gains according to rehabilitation strategy are summarized in Table 3.

**Table 3:** Comparison of functional improvements according to rehabilitation strategy Between-group comparisons were performed using the independent Student's t-test. Statistical significance was defined as p < 0.05. Δ: post-rehabilitation value − baseline value; VO₂peak: peak oxygen consumption; SV1: first ventilatory threshold

Variable	Wearable-assisted rehabilitation (n = 54)	Conventional rehabilitation (n = 67)	P-value
ΔVO₂peak (mL/kg/min)	+4.9	+5.1	0.902
ΔSV1 (mL/kg/min)	+2.02	+2.00	0.902

Overall, wearable-assisted rehabilitation achieved functional gains that were virtually identical to those obtained through conventional center-based rehabilitation.

## Discussion

The present study evaluated the impact of wearable-assisted and conventional cardiac rehabilitation on functional capacity in patients with chronic heart failure using objective CPET-derived parameters. Three principal findings emerged from our analysis. First, participation in a structured rehabilitation program was associated with substantial improvements in exercise performance. Second, these benefits were observed not only for VO₂peak but also for SV1, suggesting favorable adaptations across different domains of aerobic function. Third, wearable-assisted rehabilitation achieved functional gains comparable to those observed with conventional rehabilitation.

Exercise intolerance represents one of the defining features of chronic heart failure and remains a major determinant of reduced quality of life and adverse clinical outcomes [5,10]. In our cohort, rehabilitation was associated with a marked increase in VO₂peak, with mean values rising from 11.75 to 16.75 mL/kg/min. This finding is consistent with previous studies demonstrating the capacity of structured exercise training to improve aerobic performance in heart failure populations [11-14]. The magnitude of improvement observed in our study is clinically relevant and reflects a substantial enhancement in functional reserve. Moreover, VO₂peak is recognized as a powerful predictor of clinical outcomes in individuals with heart failure [12].

An equally important observation concerns the behavior of the SV1. While VO₂peak remains the most frequently reported CPET parameter, SV1 may better reflect functional performance during daily activities because it characterizes exercise intensity below maximal effort [1,2]. The increase in SV1 observed after rehabilitation suggests improved aerobic efficiency and delayed metabolic limitation during exercise. These adaptations likely reflect a combination of cardiovascular, muscular, and peripheral physiological responses to training [3,4].

The most notable finding of the present study was the absence of significant differences between wearable-assisted and conventional rehabilitation. Improvements in VO₂peak and SV1 were nearly identical between groups, indicating that the physiological benefits of rehabilitation were preserved despite differences in program delivery. These findings support the concept that appropriate regulation of exercise intensity may be more important than the physical location in which rehabilitation is performed. Similar observations have been reported in studies evaluating remote and hybrid rehabilitation strategies using wearable technologies and telemonitoring systems [13,14].

This observation carries particular relevance in settings where access to conventional rehabilitation remains limited. In many healthcare systems, participation in rehabilitation programs is restricted by geographical distance, transportation difficulties, limited availability of specialized centers, and economic constraints [7]. Wearable technologies offer a practical strategy to overcome some of these barriers while maintaining objective monitoring of exercise intensity [8,15,16]. Rather than replacing conventional rehabilitation, wearable-assisted programs may expand access to evidence-based exercise interventions for patients who would otherwise remain untreated, particularly in low-resource settings [15].

Beyond the short-term improvements in functional capacity observed in the present study, the growing body of evidence supporting remote and technology-assisted rehabilitation deserves consideration. The Telerehabilitation in Heart Failure Patients (TELEREH-HF) randomized trial demonstrated that hybrid telerehabilitation is feasible and safe in patients with heart failure and may contribute to sustained clinical benefits over time [17]. Furthermore, expanding access to cardiac rehabilitation has become a major international priority, particularly in low-resource settings where participation remains limited despite well-established benefits [18]. In this context, advances in remote monitoring and wearable technologies provide practical tools for supervising exercise programs, enhancing patient engagement, and facilitating the delivery of rehabilitation beyond specialized centers [19].

Certain limitations of the present study merit consideration. First, the investigation was conducted at a single institution and involved a limited patient population. The duration of follow-up was limited to the rehabilitation period, preventing the assessment of long-term clinical outcomes. In addition, hospitalization and mortality data were not evaluated. Nevertheless, the use of objective CPET-derived parameters before and after rehabilitation strengthens the physiological validity of our findings [1-4].

Future multicenter studies involving broader cohorts and extended follow-up durations are needed to establish whether wearable-assisted rehabilitation can achieve comparable long-term clinical outcomes, including reductions in hospitalization and mortality, while maintaining the functional benefits observed in the present study.

Limitations

Several limitations should be acknowledged. The study was conducted at a single institution and included a relatively modest sample size, which may limit the generalizability of the findings. The follow-up was limited to the 12-week rehabilitation period, and long-term outcomes such as hospitalization and mortality were not assessed. In addition, 22 of the 143 randomized patients (15.4%) did not complete the follow-up assessment, which may have introduced attrition bias despite the documented reasons for non-completion. Finally, the study focused mainly on CPET-derived functional outcomes and did not include multivariable analysis to assess the independent contribution of wearable-assisted rehabilitation while accounting for baseline characteristics such as NYHA class, LVEF, and medical therapy.

## Conclusions

In patients with chronic heart failure, a structured 12-week cardiac rehabilitation program was associated with significant improvements in functional capacity, demonstrated by increases in both VO₂peak and SV1. Wearable-assisted rehabilitation achieved functional gains comparable to those of conventional center-based rehabilitation, as objectively assessed by CPET. By evaluating a wearable-assisted home-based approach in a North African, resource-limited setting, this study adds to the growing evidence supporting technology-assisted cardiac rehabilitation as a potential strategy to improve access to structured exercise programs where conventional rehabilitation facilities are limited.
